# Positive or negative? The shell alters the relationship among behavioral defense strategy, energy metabolic levels and antioxidant capacity in freshwater turtles

**DOI:** 10.1186/s12983-019-0301-5

**Published:** 2019-02-13

**Authors:** Wenyi Zhang, Cuijuan Niu, Yukun Liu, Kenneth B. Storey

**Affiliations:** 1 Ministry of Education Key Laboratory for Biodiversity Science and Ecological Engineering, College of Life Sciences, Beijing Normal University, Beijing, 100875 China; 2 State Key Laboratory of Marine Environmental Science, College of Ocean and Earth Sciences, Xiamen University, Xiamen, 361102 People’s Republic of China; 3 Institute of Biochemistry and Department of Biology, Carleton University, 1125 Colonel By Drive, Ottawa, ON K1S 5B6 Canada

**Keywords:** Antioxidant capacity, Behavioral defense strategy, Freshwater turtles, Resting metabolic rate, Shell

## Abstract

**Background:**

The relationships among energy metabolic levels, behavioral and other physiological traits help to determine the trade-off of energy allocation between different traits and the evolution of life-history driven by natural selection. However, these relationships may be distinctive in selected animal taxa because of their unique traits. In the present study, the relationships among energy metabolic levels, behavioral defense strategies, and antioxidant capacity were explored in three freshwater turtle species with different shell morphologies, by assessing responses to attack, righting time, shell morphology, whole-organism metabolic rates, tissue metabolic enzyme activities and antioxidant levels.

**Results:**

The Chinese three-keeled pond turtles, *Chinemys reevesii*, showed a passive defense strategy, relatively larger shells, a higher resting metabolic rate (RMR) and higher antioxidant levels compared to the snapping turtle, *Chelydra serpentina,* or the Chinese soft-shelled turtle, *Pelodiscus sinensis.* These latter two species both showed an active defense strategy, a higher factorial aerobic scope and better muscle anaerobic metabolic capacity but relatively smaller shells, lower RMR and antioxidant capacity*.*

**Conclusion:**

Our results indicate a negative relationship between RMR and activity levels in behavioral defense strategies along small-big shell continuum among the three turtle species. We also found a positive relationship between antioxidant capacity and energy metabolism but a negative one between antioxidant capacity and activity levels in defense strategies. The present study indicated a role of turtle shell in forming unique relationship between energy metabolic levels and behaviors in freshwater turtle taxa and a possible trade-off between the maintenance of physiological homeostasis and activity levels in energy allocation.

**Electronic supplementary material:**

The online version of this article (10.1186/s12983-019-0301-5) contains supplementary material, which is available to authorized users.

## Background

Energetics and animal behaviors (e.g. predator avoidance strategies, boldness or exploration) are related to each other because both are associated with the slow-fast life-history continuum [1–3]. Thus, the relationship between energy metabolism and specific behavioral traits can show the consequences of natural selection as well as suggest the evolution of life histories and can also be expanded to link with other physiological or morphological traits [2, 4, 5]. For example, basal metabolic rate (BMR) was shown to correlate positively with animal activity levels at an interspecific level among most bird species [6–8] and also correlate positively with antioxidant capacity, an important redox balance parameter [9]; both indicated evolution along the slow-fast continuum. However, the relationship between energy metabolic levels and animal behaviors is not consistent across all animal taxa. In addition to behavioral traits, the links between energy metabolism and physiological or other traits also matter because they reflect the principle of energy allocation to other different functions based on the activity levels [3]. Natural selection generally acts most directly on behaviors and/or energy metabolism, but much less directly on lower-level physiological or other traits [10, 11]. Thus, exploring the relationship among energy metabolic level, behavioral and other traits in specific animal taxa can help our understanding of how interspecific variations are maintained among related species by revealing how selection affects behaviors, energy metabolism and other traits and also reveal which traits are dominant therein.

The unique traits of freshwater turtles raise questions about their evolution, behaviors and physiology. They have peculiar characteristics as their unique shell [12] and their long lifespan [13]. The shell offers extra protection when turtles are attacked by predators or encounter environmental stressors but shell formation would incur extra energy costs during development and growth. Thus, the presence of the shell may result in special relationship between energy metabolic levels and turtle behaviors under the selection of predation or environmental stress, which may further affect other physiological traits. Among freshwater turtle species, interspecific variations in shell morphology, behavioral traits (e.g. righting response) and physiological traits (e.g. antioxidant capacity or immunity) have been widely observed [14–19]. How these variations were shaped, maintained and linked may be explained by the relationship between energy metabolism and these traits although this relationship is not yet well understood.

**Fig. 1:**
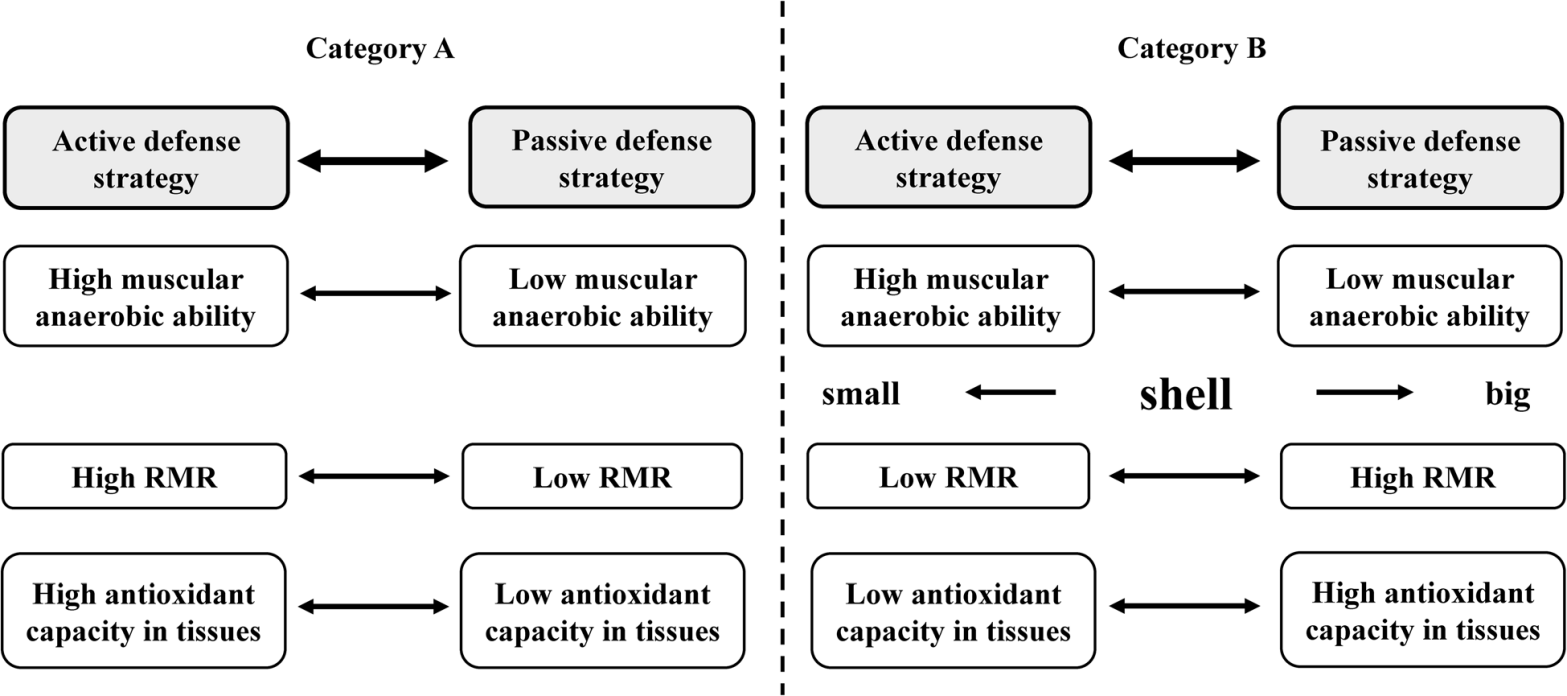
The relationships among energy metabolic capacity, behavioral defense strategies and antioxidant capacity observed in birds **a**, and predicted for freshwater turtles with the inclusion of shell morphology **b**

The aim of our study was to explore the relationships among energy metabolism, behavioral and physiological traits in freshwater turtles and how the shell participates in these relationships. We selected three freshwater turtles living in similar habitats for the study: the Chinese three-keeled pond turtle *Chinemys reevesii* that has a hard shell, the Chinese soft-shelled turtle *Pelodiscus sinensis* with a soft shell and the snapping turtle *Chelydra serpentina* with a small plastron [20–22]. Among behavioral traits, behavioral defense strategies were chosen because they link with short-term fitness and can be affected by natural selection of predation or environmental stress. Two main defense strategies are observed in freshwater turtles: the active and the passive. The active strategy showed high activity level along with rapid behavioral response (e.g. escape or bite back) to attack or stress and efficient anaerobic metabolism [23, 24]. The passive strategy includes a low activity level along a defensive strategy of retracting head, legs and tail into the shell for a long time [23]. Direct energy costs of active behaviors can be avoided but an extra energy investment into the shell may be necessary for the passive strategy. Thus, we predicted a negative relationship between activity levels used in defense strategies and energy metabolic capacity along a small-big shell continuum (see Fig. 1b), which may be different from that seen in birds (Fig. 1a). At the same time, the present study also assessed on antioxidant capacity because it links with long-term fitness and stress tolerance [25, 26]. In the passive strategy, the use of long-term hiding in the shell may promote high constitutive antioxidant capacity. Thus, we also predicted a negative relationship between activity levels in defense strategies and antioxidant capacity in freshwater turtles (Fig. 1b) and a further positive correlation between energy metabolic capacity and antioxidant capacity, which is similar to that seen in birds (shown as Fig. 1a). In the present study, different behavioral defense strategies of the three species were distinguished by variation in righting time and direct responses to attack. Whole-organism metabolic rate and tissue metabolic enzyme activities were employed to indicate energy metabolic capacity as well as tissue total antioxidant capacity (TAC) and antioxidant enzyme activities to indicate antioxidant capacity. Shell morphology was assessed by measuring carapace height/width ratio and plastron/carapace flat area ratio.

## Methods

### Animals holding and experimental process

Chinese soft-shelled turtles, *P. sinenesis* (*n* = 15, mass = 112.6 ± 8.3 g), were obtained from a turtle hatchery facility (Yutian County, Hebei province, PR China). *C. reevesii* (n = 15, mass = 108.2 ± 5.9 g) and *C. serpentina* (n = 15, mass = 148.5 ± 5.2 g) were obtained from an aquarium market (Beijing Guanyuan market, PR China). All turtles were raised in tanks (70 × 50 × 40 cm with water depth of about 6 cm) at 26 ± 1 °C (a suitable temperature for the feeding and growth of all three species) for at least 6 weeks. All turtles were given commercial feed daily. Photoperiod was kept at 12 L/12 D.

After acclimation, all turtles were assessed for (a) behavioral traits: righting time (RT) and response to attack (RTA), (b) morphological traits: carapace height/width ratio (H/W ratio), plastron/carapace flat area ratio (P/C ratio), and (c) metabolic traits: RMR, maximum metabolic rate (MMR), net aerobic scope (NAS) and factorial aerobic scope (FAS). Then turtles were transferred back to their original tanks for another two weeks. Afterwards, all individuals of each species were divided into two groups. One group was defined as the control group without treatment (*n* = 7). The other group, which was defined as the electrical stimulated group (ESG, *n* = 8), experienced forced movement by electrical stimulation (30-50 V, 20 hz, 10 msec, 5 min or to exhaustion) [27–29]. After treatment, all turtles were sacrificed to excise liver, heart and skeletal muscle of the left forelimb. Liver and heart were weighed to provide liver index and heart index. All tissues were frozen in liquid nitrogen immediately and stored at − 80 °C. Activities of selected enzymes were measured. These included (a) energy metabolism related mitochondrial respiratory enzymes: succinate dehydrogenase (SDH), respiratory complex III (C3), cytochrome c oxidase (CO) and lactate dehydrogenase (LDH), and (b) antioxidant enzymes: TAC, superoxide dismutase (SOD) and catalase (CAT). Levels of lactic acid (LA) and ascorbic acid (AA) in tissues were also measured.

### Behavioral traits

Righting time reflects the response to being overturned and the hiding time until the predator leaves [14, 30]. The response to attack reflects the choice between different behavioral defense strategies of freshwater turtles when disturbed. The water temperature for all behavioral assays was maintained at 26 °C and a 30 min acclimation period to testing area was applied for each turtle before treatments. For the righting time survey, each turtle was placed in one thermostatic chamber (diameter = 20 cm, water depth = 5 cm). After acclimation, turtles were turned upside down in the chamber to start the test. The righting process was recorded with a camera to the nearest second starting from the moment that the turtle was placed upside down to the moment that the animal righted itself completely [31]. For response to attack, a similar method was used as described by Chang et al. [32]. Each turtle was placed in one tank (140 × 60 × 70 cm, water depth = 5 cm) and was tapped on the carapace with a tweezer from a hidden position. The immediate response was recorded and graded with the following criteria: (1) run away, (2) stay still, (3) turn to attack, or (4) retraction into the shell. Each individual was tested twice, as in previous related studies [14, 31].

### Shell morphology, liver and heart index

Maximum carapace height and carapace straight width were measured with a digital caliper (Pro skit, PR China) to the nearest millimeter. According to the model of Domokos and Várkonyi [15], H/W ratio of turtles was used to judge if the turtle species belonged to “flat turtles” (H/W under approx. 0.6), “medium turtles” (H/W between 0.6 and 0.8) or “tall turtles” (H/W above approx. 0.8) groups. Flat areas of carapace and plastron were measured with a camera and assessed using image analysis software (Digimizer, Belgium). The P/C ratio was used to compare the degree of shell coverage of the three turtle species. Liver index and heart index were calculated as the ratio of tissue wet weight to fresh animal body weight.

### Metabolic rates and aerobic scope

RMR of each turtle was measured in air at 26 °C using an airtight chamber (diameter = 20 cm, height = 6.5 cm) and oxygen sensor (FireStingO2, Pyro science, Germany). Turtles were fasted for 24 h and kept still in the chamber. Each individual was acclimated to the experimental environment for 30 min with the cover off. Then the cover was closed tightly, the survey was started and the change in oxygen percentage (%) over 30 min was recorded. To measure the MMR, turtles were forced to run for 5 min or until exhaustion in response to electrical stimulation (30-50 V, 20 hz, 10 msec). Then the turtle was quickly placed in the chamber to measure the change in oxygen percentage (%) over the following 30 min. The change in oxygen percentage inside the chamber during the initial 100 s was chosen to calculate the MMR. The air volume was measured by filling the chamber with water in the presence of the animal and then measuring the volume of water to the nearest milliliter. FAS (defined as the MMR/RMR ratio) and NAS (defined as difference between MMR and RMR) were calculated [33].

### Biochemical assays

After sampling, tissues were homogenized in phosphate buffered solution (9 g/L NaCl, 726 mg/L Na_2_HPO_4_-7H_2_O, and 210 mg/L KH_2_PO_4_, pH 7.2) for biochemical assays. Reagent kits (Nanjing Jiancheng, PR China) were used to measure the activities of SDH, C3, CO, SOD, CAT, LDH, TAC and LA level according to the instruction manual for each kit.

Gradient centrifugation was employed to isolate mitochondria from tissue homogenates for the SDH, C3 and CO activity assays. To do this, homogenates were centrifuged at 1000 g for 10 min, then supernatants were removed and recentrifuged at 12000 g for 15 min, followed by retrieving pellets, that were then washed and resuspended (all steps were done at 4 °C). SDH activity was measured using a 2,6-dichlorophenolindophenol (DCPIP) reduction reaction. The DCPIP reduction speed was determined by the change in absorption value at 600 nm [34]. C3 activity was measured using a reaction in which the C3 complex reduced oxidized cytochrome c to the reduced form in the presence of a hydrogen donor and the increase in absorbance at 550 nm was measured for 2 min [35]. CO activity was measured using a reaction that converted cytochrome c to its oxidized form and the decrease in absorbance value at 550 nm was measured for 1 min [35].

Portions of each tissue homogenate were centrifuged at 4500 g and 4 °C for 10 min to provide supernatants for antioxidant enzyme activity assays, LDH activity and LA content. TAC was measured by determining the absorption value at 520 nm when Fe^3+^ was reduced to Fe^2+^ in the presence of antioxidants [36]. SOD activity was measured using a cytochrome c reduction inhibition reaction in a xanthine-xanthine oxidase system and the degree of inhibition was measured by the absorption value at 550 nm [37]. CAT activity was measured by determining the rate of H_2_O_2_ decomposing in 1 g protein per second with the change of absorption measured at 405 nm. LDH activity was measured using the reverse reaction in which LDH catalyzes the conversion of lactate to pyruvate using NAD^+^ as the hydrogen donor. Then 2,4-dinitrophenylhydrazine was used to determine pyruvate product levels, monitored by absorption change at 440 nm. LA content was measured following the method of Buttery et al. [38]. LA was transformed to pyruvate with the generation of NADH, which reacted with phenazine methosulfate; then the products reduced p-iodonitrotetrazolium to its reduced form which was assessed by the absorption value at 530 nm. Total protein content was measured according to the Coomassie blue dye-binding method with bovine serum albumin as the standard [39].

We employed the method described in Chen et al. [40] to measure tissue AA content, which is an essential antioxidant in turtles [41, 42]. Tissues were homogenized with 15% cold-phosphoric acid at a ratio of 1:19 (*w*/*v*) and then homogenates were centrifuged at 20,000 g for 20 min. The supernatants were used for high performance liquid chromatography (HPLC) analysis with electrochemical detection (Waters 2695/2487, USA) and a 5 μm analytical column (4.6 × 250 mm, Aglient, USA). Injection volume was 20 μL, flow rate was 1 mL per min and ultraviolet detection was set as 243 nm.

### Statistics

The hierarchical clustering algorithm was generated with all data according to a Euclidean method. The clustering analysis and the heatmap were conducted with R v. 3.5.0 (Development Core Team 2018). Then, all data were checked for normality by the Kolmogorov-Smirnov test and homogeneity of variance. For metabolic rates, a general linear model was employed to conduct interspecies comparation with mass as covariate. For enzyme activities and LA level, intraspecific differences between the control group and the treatment group (ESG) were assessed via a t-test. If no significant difference between the two groups was found, data of the two groups were combined for interspecific comparison. Interspecific variations in all parameters, except metabolic rates, were detected using one-way ANOVA followed by Tukey HSD post hoc test, or Kruskal-Wallis test followed by Mann-Whitney U post hoc test for data that did not fit the check for normality or variance homogeneity. *P* < 0.05 was set as the significance level. The correlations among different morphological and physiological parameters were analyzed using Pearson’s correlation analysis, while the correlations between behavioral parameters, which are discrete variables, and others were analyzed using Spearman’s correlation analysis.

## Results

**Fig. 2:**
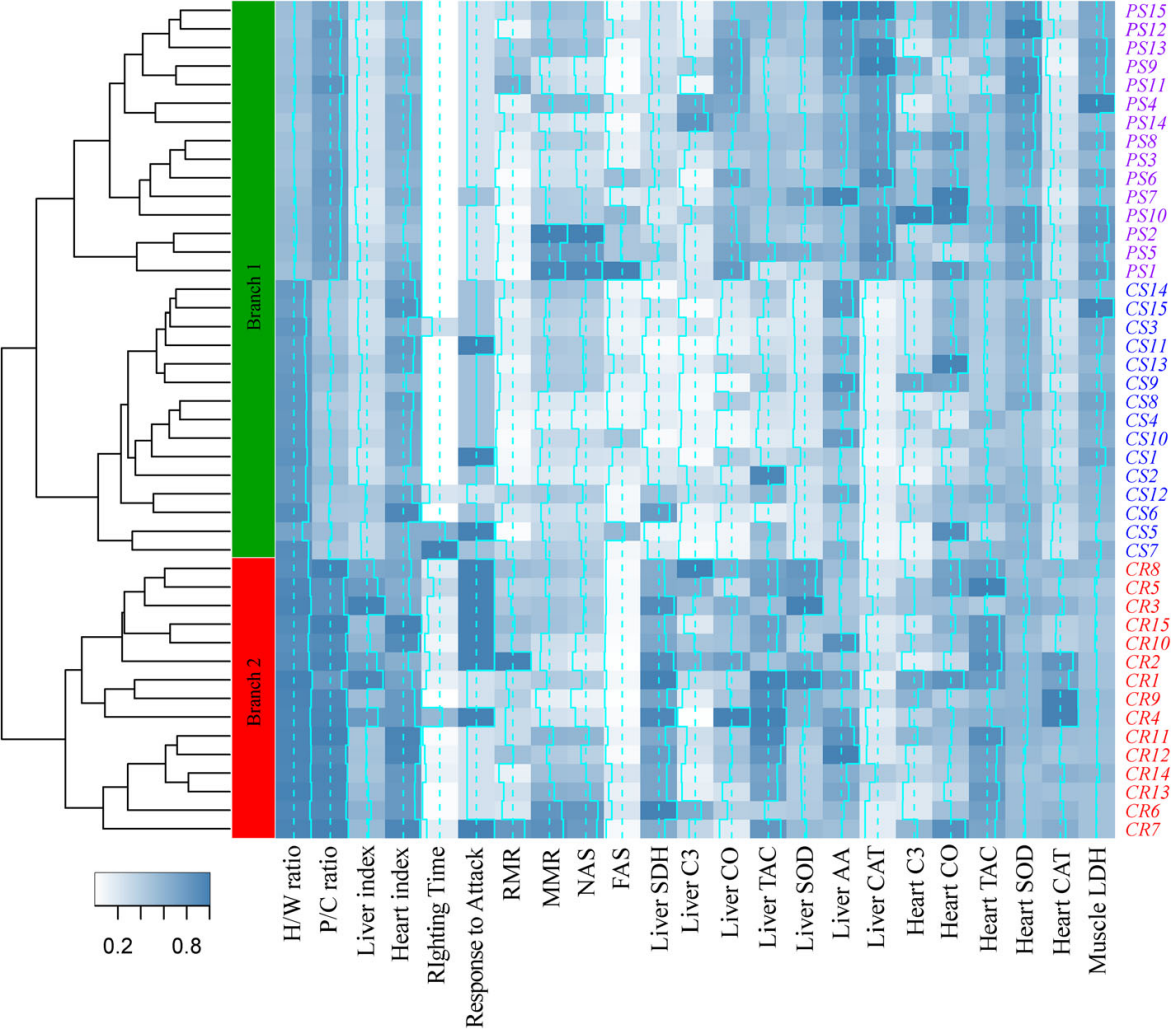
Heatmap showing clustering results of all individuals based on all parameters measured. For each parameter, the data were standardized according to the maximum value within the group. In each block, the dashed line indicates the 0.5 value and the trace line indicated the observed value. The tree on the left indicates two branches from three freshwater turtles. The green branch 1 includes *P. sinensis* and *C. serpentina* and the red branch 2 includes all *C. reevesii*

**Fig. 3:**
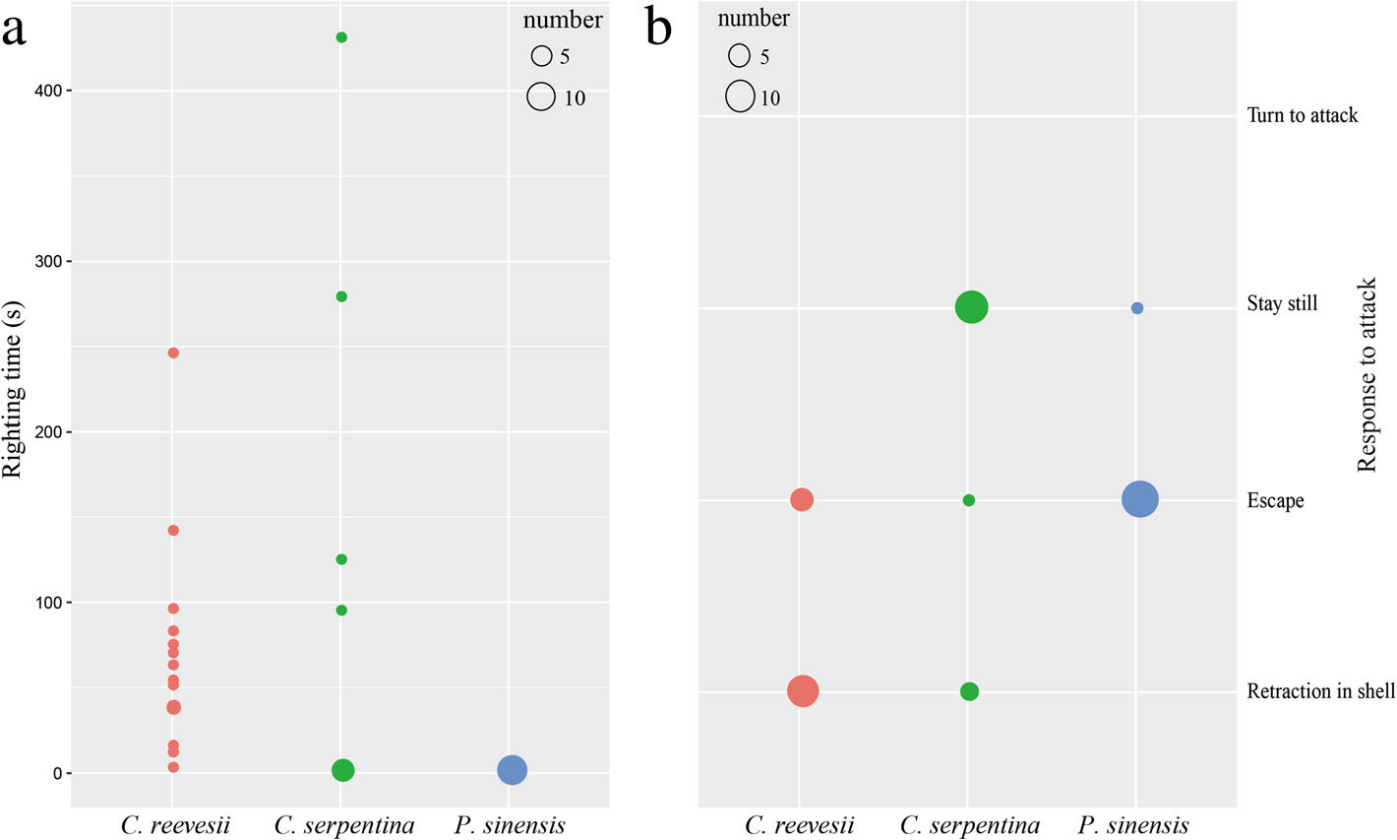
The righting time **a** and response to attack **b** of three freshwater turtle species. The size of circle means the number of animals

**Fig. 4:**
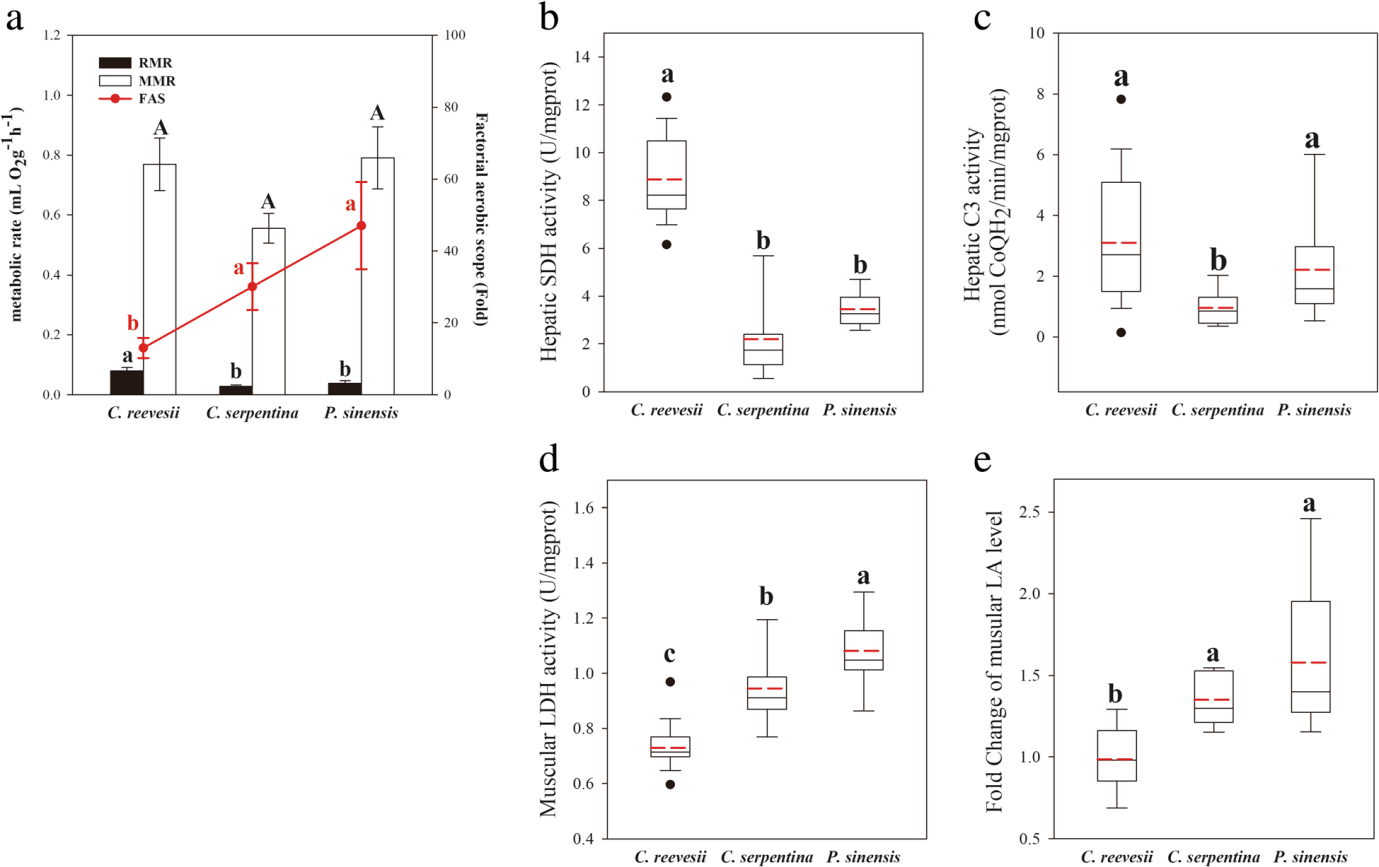
Energy metabolic index **a**, hepatic SDH activity **b**, hepatic C3 activity **c**, muscular LDH activity **d** and fold change in muscular LA level **e** in three freshwater turtle species. Superscripts without common letters denote significant difference (*P* < 0.05)

**Fig. 5:**
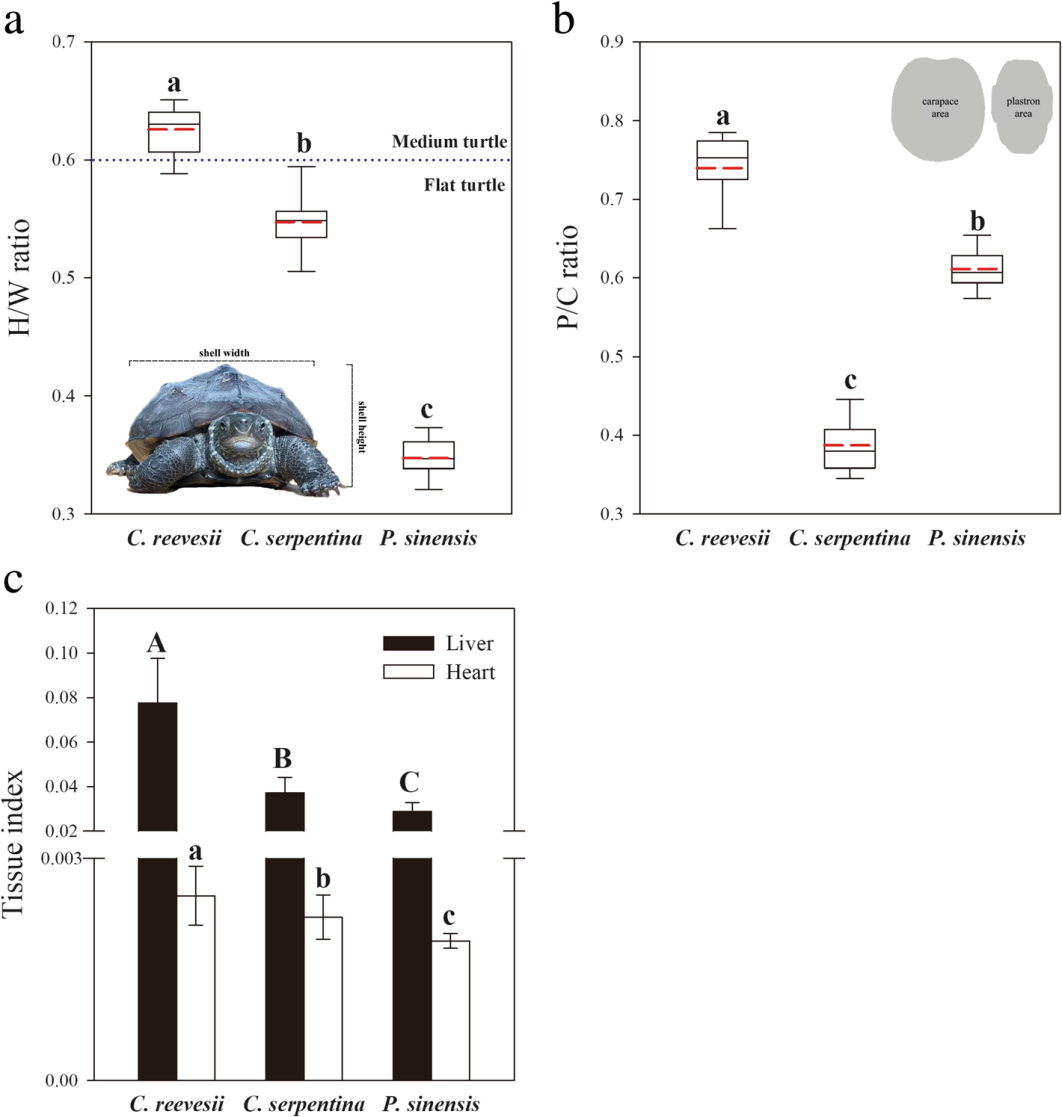
The carapace height/width ratio **a**, plastron/carapace flat area ratio **b**, liver index and heart index **c** in three freshwater turtle species. Superscripts without common letters denote significant difference (*P* < 0.05). In **c**, the data are presented as mean ± SE

**Fig. 6:**
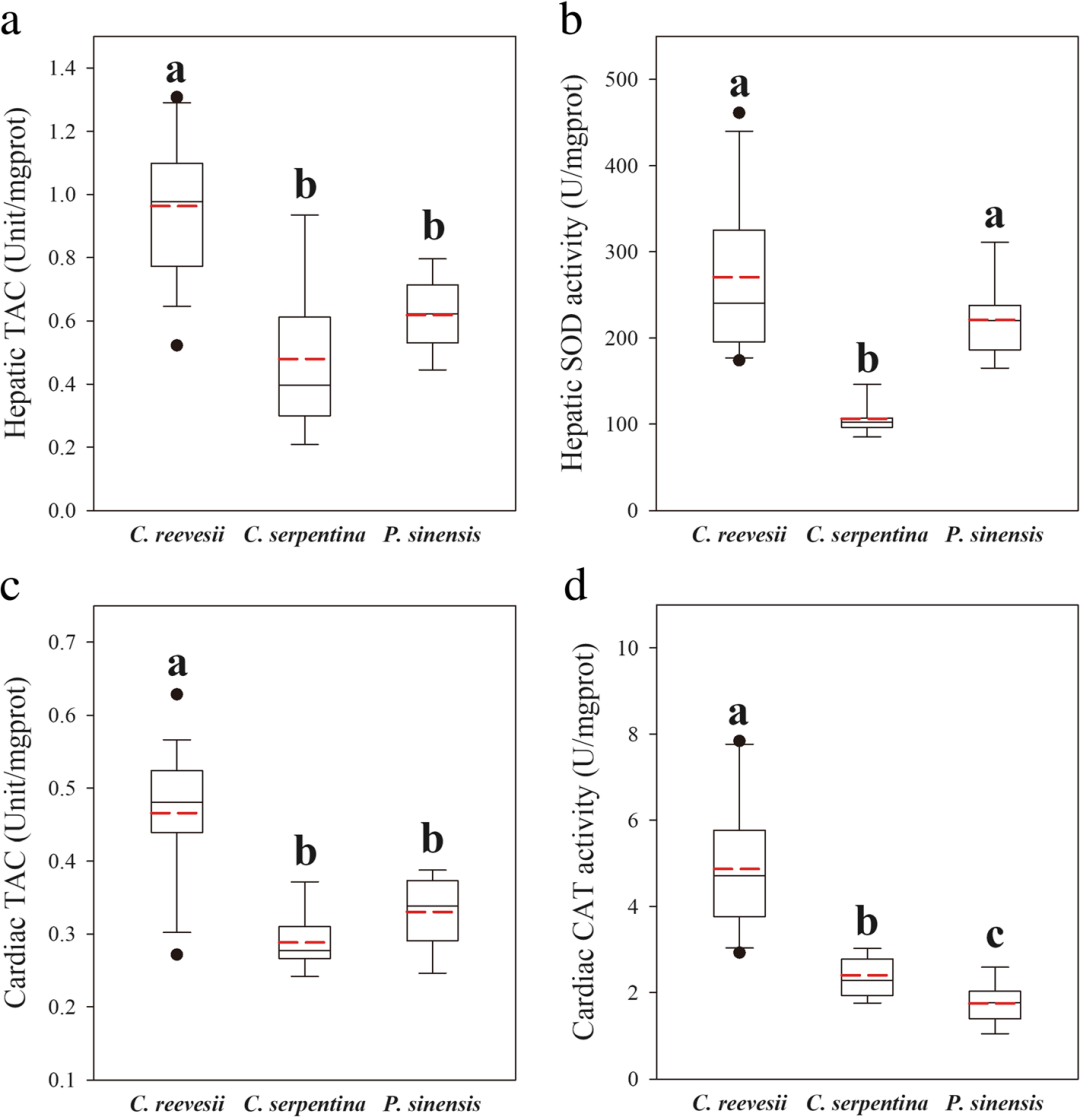
Hepatic TAC **a**, hepatic SOD activity **b**, cardiac TAC **c** and cardiac CAT activity **d** in three freshwater turtle species. Superscripts without common letters denote significant difference (*P* < 0.05)

Clustering results showed that the 45 turtles can be divided into two groups according to all parameters measured (Fig. 2). One group included all individuals of *P. sinensis* and *C. serpentina*. The other one included all *C. reevesii* individuals. Detailed results were shown in Figs. 3, 4, 5 and 6 or Additional file 2: Figure S1, Additional file 3: Figure S2 and Additional file 4: Figure S3.

### Behavioral traits

In the righting test, *C. reevesii* spent the longest time whereas *P. sinensis* spent the shortest time in righting themselves (RT_*C.reevesii*_ = 66.6 ± 16.0 s, RT_*C. serpentina*_ = 63.3 ± 33.0 s and RT_*P. sinensis*_ = 1.3 ± 0.2 s, χ^2^ = 22.746, df = 2, *P* < 0.001, Fig. 3a). The three turtle species also showed different responses to attack (χ^2^ = 16.941, df = 2, *P* < 0.001, Fig. 3b). More than half of *C. reevesii* retracted into shell whereas the others ran away. All *P. sinensis* except one ran away when faced with attack and most *C. serpentina* stayed still (Fig. 3b).

### Metabolic rates, aerobic scopes and metabolic enzyme activities

*C. reevesii* showed the highest RMR compared with the other two species (F_2,41_ = 10.438, *P*_species_ < 0.001, *P*_mass_ = 0.259 and *P*_interaction_ = 0.551, Fig. 4a). Maximum metabolic rate (MMR) of these three turtle species showed no difference (F_2,41_ = 0.169, *P*_species_ = 0.845, *P*_mass_ = 0.020 and *P*_interaction_ = 0.886, Fig. 4a). Factorial aerobic scope (FAS) of both *C. serpentina* and *P. sinensis* were higher than that of *C. reevesii* (F_2,41_ = 4.592, *P*_species_ = 0.016, *P*_mass_ = 0.427 and *P*_interaction_ = 0.978, Fig. 4a).

In the liver, SDH activity of *C. reevesii* was higher than the other two species (F_2,42_ = 70.002, *P* < 0.001, Fig. 4b). Hepatic C3 activities of *C. reevesii* and *P. sinensis* were higher than that of *C. serpentina* (χ^2^ = 10.758, df = 2, *P* = 0.005, Fig. 4c). *P. sinensis* had the highest muscular LDH activity, while that of *C. reevesii* was the lowest (F_2,42_ = 25.267, *P* < 0.001, Fig. 4d). Forced movement only induced muscle LA accumulation in *C. serpentina* and *P. sinensis* but not in *C. reevesii* (*P*_*c. serpentina*_ < 0.001; *P*_*p. sinensis*_ = 0.045 and *P*_*c. reevesii*_ = 0.722, Fig. 4e).

### Morphological traits, liver and heart index

*C. reevesii* showed the highest carapace H/W ratio (F_2,42_ = 611.948, *P <* 0.001), P/C ratio (F_2,42_ = 382.29, *P* < 0.001), liver index (χ^2^ = 32.949, df = 2, *P* < 0.001) and heart index (F_2,40_ = 14.24, *P* < 0.001) among the three turtle species (Fig. 5). *P. sinensis* showed a higher P/C ratio but lower values for other morphological parameters than those of *C. serpentina* (Fig. 5). Both H/W ratio and P/C ratio positively correlated with RMR among the three species (RMR vs. H/W ratio: r = 0.322, *P =* 0.031; RMR vs. P/C ratio: r = 0.510, *P* < 0.001).

### Antioxidant capacity

In hepatic antioxidant defense, *C. reevesii* showed the highest TAC among the three species (F_2,42_ = 20.515, *P* < 0.001, Fig. 6a). The SOD activity in the liver of *C. serpentina* was the lowest among the three turtles (χ^2^ = 29.4123, df = 2, *P* < 0.001, Fig. 6b). In the heart, *C. reevesii* showed the highest TAC as well as CAT activity (TAC: χ^2^ = 23.279, df = 2, *P* < 0.001, Fig. 6c; CAT: χ^2^ = 31.529, *P* < 0.001, Fig. 6d).

### Correlation among behavioral traits, RMR, metabolic and antioxidant capacity

The righting time correlated positively with shell morphological traits, liver index, RMR and TAC in the liver and heart of the three turtle species (Table 1). Response to attack only correlated positively with H/W ratio and liver index (Table 1). Both behavioral traits correlated negatively with muscle LDH activity (Table 1).

**Table 1:** Spearman’s correlation between behavioral and primary morphological parameters as well as physiological parameters (*n* = 45)

	H/W ratio	P/C ratio	Liver index	RMR	Hepatic TAC	Cardiac TAC	Muscle LDH
Righting time							
Coefficient	0.648	0.357	0.701	0.412	0.429	0.415	−0.728
Sig.	< 0.001	0.016	< 0.001	0.005	0.003	0.005	< 0.001
Response to attack							
Coefficient	0.377	−0.056	0.405	0.16	−0.013	−0.067	− 0.400
Sig.	0.011	0.714	0.006	0.294	0.932	0.663	0.007

**Fig. 7:**
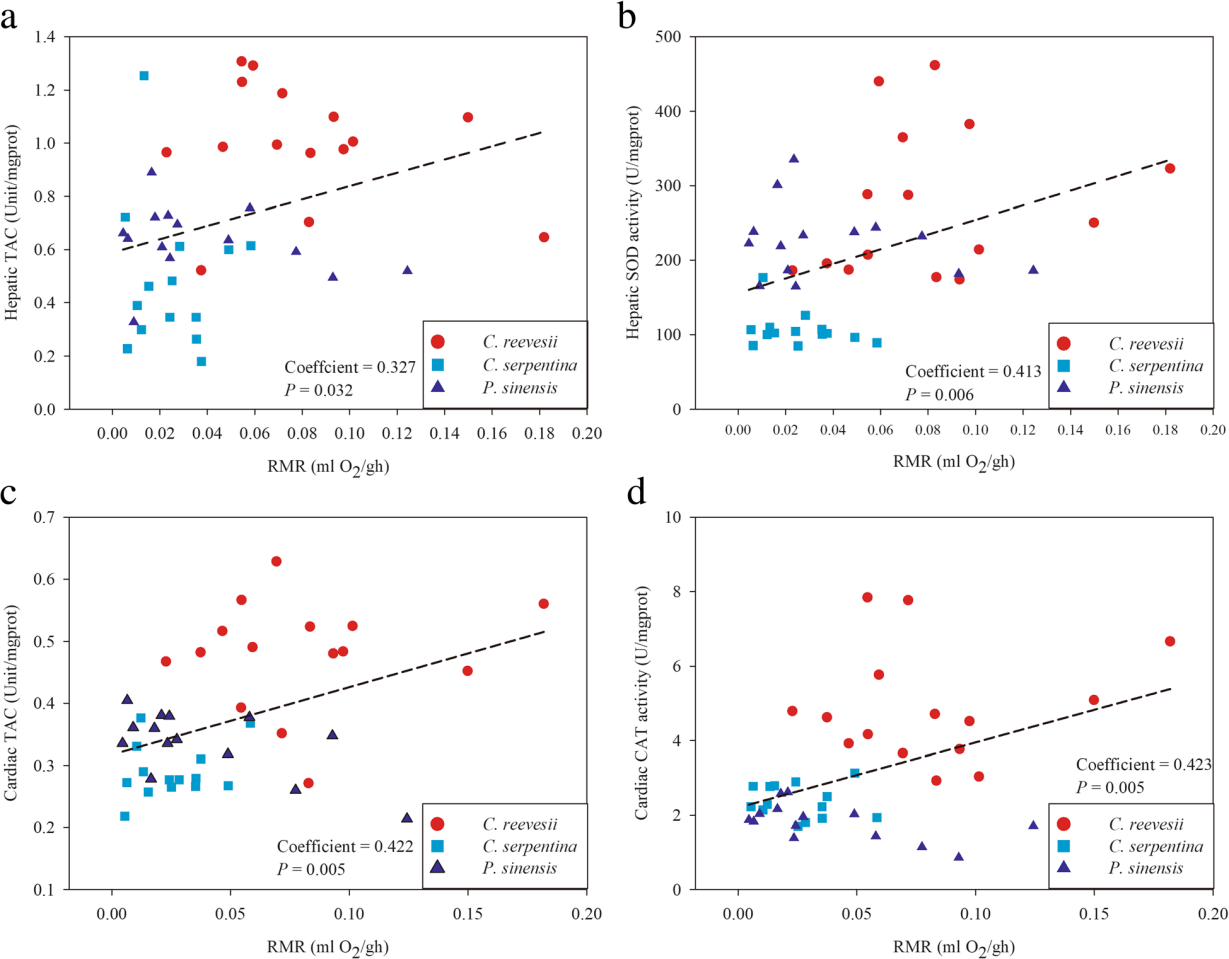
Correlation between RMR and antioxidant enzyme activities. **a** positive correlation between hepatic TAC and RMR, **b** positive correlation between hepatic SOD activity and RMR, **c** positive correlation between cardiac TAC and RMR, **d** positive correlation between cardiac CAT activity and RMR

Of all antioxidant components measured in the three species, hepatic SOD activity correlated positively with TAC in the liver (r = 0.524, *P* < 0.001) and CAT activity positively correlated with TAC in the heart (r = 0.482, *P* = 0.001). Furthermore, these parameters positively correlated with RMR (hepatic TAC vs. RMR: r = 0.330, *P* = 0.027, Fig. 7a; hepatic SOD vs. RMR: r = 0.400, *P* = 0.006, Fig. 7b; cardiac TAC vs. RMR: r = 0.425, *P* = 0.005, Fig. 7c; cardiac CAT vs. RMR: r = 0.426, *P* = 0.003, Fig. 6d, respectively).

## Discussion

Relationships among energy metabolic levels, behavioral and other traits link with energy constraints and trade-offs driven by natural selection. In the present study, as we predicted, a negative relationship was observed between energy metabolic levels, antioxidant capacity and activity levels in behavioral defense strategies along small-big shell continuum, indicating two possible branches in freshwater turtle taxa driven by the predation or environmental stress (Fig. 2).

### Negative relationship between metabolic level and activity levels in defense strategy linked with shell morphology

The behavioral assays showed that a passive defense strategy was used by *C. reevesii* whereas an active defense strategy was used by *C. serpentina* and *P. sinensis* (Fig. 3). Both the long righting time and the behavioral response, retraction into the shell, showed by more than half of the *C. reevesii* individuals indicated that *C. reevesii* tended to hide in their shell and endure the disturbance until a confirmation of safety was achieved. However, the strategy of *P. sinensis* was the complete opposite. *P. sinensis* showed rapid righting and escape behaviors when disturbed, indicating high activity levels in its defense strategy (Fig. 3). Interestingly, *C. serpentina* showed a short righting time and did not show behavioral response when disturbed (Fig. 3). In Dodd and Brodie’s report [21], *C. serpentina* moved to keep facing the attacker with lifting its shell and biting behavior, but animals did not retract into their shell when they encountered intense attack or were disturbed. Thus, *C. serpentina* tend to be an active defense strategy user with a high activity level in response to intense stress. The active defense strategy of *C. serpentina* and *P. sinensis* was also linked with the higher FAS value and better muscular anaerobic capacity in both two species (Fig. 4a, d and e). The higher FAS value reflects a higher oxygen debt bearing capacity related to RMR [43]. High FAS has been observed in fish and lizard species that frequently experience intense combat as the consequence of physiological adaptation to high activity level [44–46]. Similarly, better muscular anaerobic capacity, indicated by higher muscle LDH activity and quicker LA accumulation in *C. serpentina* and *P. sinensis* than that in *C. reevesii* (Fig. 4d and e), was also shaped by high activity level in life history and has been observed in animals that are good at diving, foraging and escaping [47, 48].

The three freshwater turtles also varied in their resting metabolic rates (RMR) with a higher RMR of *C. reevesii* versus lower RMR in *C. serpentina* and *P. sinensis* (Fig. 4a). The higher RMR of *C. reevesii* indicated higher energy investment in maintenance [49] and links with relatively larger organs, liver and heart, related to energy conversion or supply (Fig. 5c). In turn, the bigger liver also contributes to the higher RMR because the liver usually shows high mass-specific metabolic rates and can account for a large part of whole-organism metabolic rate [3]. In addition, a higher energy metabolic capacity in *C. reevesii* can also be supported by higher hepatic SDH and C3 activities (Fig. 4b and c). Mitochondria consume almost 90% of cellular respiration to provide ATP to power cell functions [50] and this process depends on the SDH and C3 activities [51, 52]. Interestingly, a positive correlation was observed between righting time and RMR among the three species (Table 1), indicating that the species with the higher RMR showed a more passive response when being placed upside down. Thus, as we predicted in Fig. 1, *C. reevesii* showed a passive behavioral defense strategy while a higher RMR than the other two turtle species, which showed an active strategy and lower RMR.

The reason for the negative relationship between RMR and activity level in the behavioral defense strategies of these turtles might be their unique morphological trait, the shell. Our results indicated clear difference among the shell morphology of the three species and relationship among shell morphologies, behavioral traits and RMR. The shell of *C. reevesii* was higher (greater H/W ratio, Fig. 5a) and had a relatively bigger plastron (higher P/C ratio, 5B) than the other two species, which may link with the passive defense strategy (Table 1). A higher shell can offer better protection for turtles against attack from predators and protect them during hiding in shell [53] while a flatter shell, such as that of *C. serpentina* or *P. sinensis*, is advantageous for swimming or digging and benefits their survival in active defense strategy [15, 54]. Similarly, the larger plastron can offer a larger radius of protection for the ventral side of turtles when they are overturned. Thus, the variation in shell morphology matches the different behavioral defense strategies of turtle species and can also be observed in other animals that similarly carry a similar shell, such as land snails [55]. At the same time, shell morphology also linked with RMR among the three turtle species with a positive correlation (RMR vs. H/W ratio: r = 0.322, *P =* 0.031; RMR vs. P/C ratio: r = 0.510, *P* < 0.001), indicating a greater energy cost to support bigger shells. The extra energy cost may represent both the energy required to grow and maintain the shell and the energy debt involved in the daily activity carrying the bigger shell. In fact, in the righting response, turtle species with a higher shell need less energy investment for their self-righting than the species with flat shell [15, 56]. Thus, the energy investment in shell maintenance or structure may be an energy budget in several turtle species, which can be reduced to compensate for the more energy-demanding activity in other species, and promote a unique trade-off of energy allocation between activity levels for behavioral defense strategies and shell morphology.

### Positive relationship between antioxidant capacity and metabolic levels linked with different defense strategies

Good antioxidant defenses are important for all animals to deal with oxidative stress and various chemical and xenobiotic stresses. Among freshwater turtles, our results indicated a higher tissue antioxidant capacity along with higher energy metabolic capacity (Figs. 6 and 7). On one hand, a higher RMR means a higher energy cost of self-maintenance and, in the present study, a possible cost is the investment in antioxidant defense. On the other hand, a higher RMR also may result in more reactive oxygen species (ROS) production, thereby promoting a higher tissue antioxidant capacity [57]. For example, hepatic SOD activity correlated with the mitochondrial energetics, probably to eliminate superoxide radicals (O_2_^−^) generated in mitochondria in association with high rates of aerobic respiration among the three species (Additional file 3: Figure S2).

The variation in antioxidant capacity also linked with different behavioral defense strategies (Table 1). The passive defense strategy of *C. reevesii* may result in animals taking refuge in an adverse environment, such as hiding underwater to escape predators but without access to air to breathe. There turtles can experience hypoxia/anoxia stress which is associated with excessive ROS generation in animal tissues [26]. A high constitutive antioxidant capacity favors the maintenance of redox balance without an extra energy investment when animals face with environmental stress frequently. Thus, high antioxidant capacity could be an important physiological adaption to the passive behavioral defense strategy of freshwater turtles, such as *C. reevesii*.

### Trade-off between behavioral activities and physiological homeostasis

The relationships among energy metabolism, behavioral traits and other traits reflect trade-offs of energy allocation, which is driven by natural selection (Engqvist et al. 2015; Houston and Mcnamara 1989; Réale et al. 2010; Reznick et al. 2000). Our results provided evidence of the special negative correlation between energy metabolism, antioxidant capacity and behavioral activity levels in different defense strategies of freshwater turtles (as shown in Fig. 1b). These relationships link with the energy allocation trade-off between maintenance of physiological homeostasis (big shell, high RMR and antioxidant defense) and activity levels (high FAS and efficient muscular anaerobic capacity) (Cohen et al. 2008; Galliard et al. 2013). Physiological homeostasis can be considered as less variation in physiology over time. Repeated regulation of homeostasis is costly due to the expense of adjusting all physiological processes and producing molecules involved in homeostatic regulation (Cohen et al. 2008). Among freshwater turtle species, the size and mineralization of the shell has been shown to be positively correlated with protection against predator attack or environmental stresses [58, 59]. At the same time, a high constitutive antioxidant capacity can maintain redox balance without extra energy cost in transcription and translation of antioxidant proteins when animals encounter predator attack or environmental stresses [60, 61]. Thus, some turtle species, such as *C. reevesii*, invest much energy in building their shell and in high constitutive antioxidant levels while showing a passive endurance strategy under stress or attack (Fig. 2 Branch 2). This strategy further avoids frequent fluctuations in physiological processes such as energy metabolism or redox balance. Oppositely, other turtle species, including *C. serpentina* and *P. sinensis*, show a high investment in behavioral defense strategies and are good at muscle anaerobic mechanism (Fig. 2 Branch 1). They may thus form shells with lower protection and lower constitutive tissue antioxidant capacity which both can be related to a relatively lower energy investment in maintenance. However, they may benefit from their strategy because immediate and active defense responses to extrinsic factors may cause greater adaptability to a changing environment.

## Conclusions

The present study assessed the relationships among energy metabolism, behavioral and physiological traits in three freshwater turtle species. Our results indicated a negative relationship between energy metabolic levels and activity levels in behavioral defense strategies, which is shaped by the extra energy investment needed to support shell morphology. We also found an interspecific variation in antioxidant capacity promoted by both energy metabolic levels and behavioral defense strategies. A possible trade-off of energy allocation between the maintenance of physiological homeostasis and animal activity levels can be inferred under the selective pressures of predation and environmental stress.

## Additional files

Additional file 1: Data and correlation analysis for all parameters used in the present study. (XLSX 34 kb)

Additional file 2: **Figure S1.** Hepatic CO activity (A), cardiac C3 activity (B) and cardiac CO activity in three freshwater turtle species. Superscripts without common letters denote significant difference (*P* < 0.05). (TIF 775 kb)

Additional file 3: **Figure S2.** Hepatic CAT activity (A), hepatic AA level (B) and cardiac SOD activity (C) in three freshwater turtle species. Superscripts without common letters denote significant difference (*P* < 0.05). (TIF 776 kb)

Additional file 4: **Figure S3.** The correlation between parameters measured: hepatic SDH activity and RMR (A), hepatic C3 activity and RMR (B), hepatic SOD activity and hepatic SDH activity (C), hepatic SOD activity and hepatic C3 activity (D), hepatic SOD and hepatic CO activity (E), cardiac SOD activity and cardiac C3 activity (F). (TIF 870 kb)

## Abbreviations

AA: Ascorbic acid
BMR: Basal metabolic rate
C3: Respiratory complex III
CAT: Catalase
CO: Cytochrome c oxidase
FAS: Factorial aerobic scope
H/W ratio: Carapace height/width ratio
HPLC: High performance liquid chromatography
LA: Lactic acid
LDH: Lactate dehydrogenase
MMR: Maximum metabolic rate
NAS: Net aerobic scope
P/C ratio: Plastron/carapace flat area ratio
RMR: Resting metabolic rate
RT: Righting time
RTA: Response to attack
SDH: Succinate dehydrogenase
SOD: Superoxide dismutase
TAC: Total antioxidant capacity
