# Supplementary figures and images for: Positive or negative? The shell alters the relationship among behavioral defense strategy, energy metabolic levels and antioxidant capacity in freshwater turtles
Source: Front Zool. 2019 Feb 13;16:3. doi: 10.1186/s12983-019-0301-5 (PMC6375210; doi:10.1186/s12983-019-0301-5)

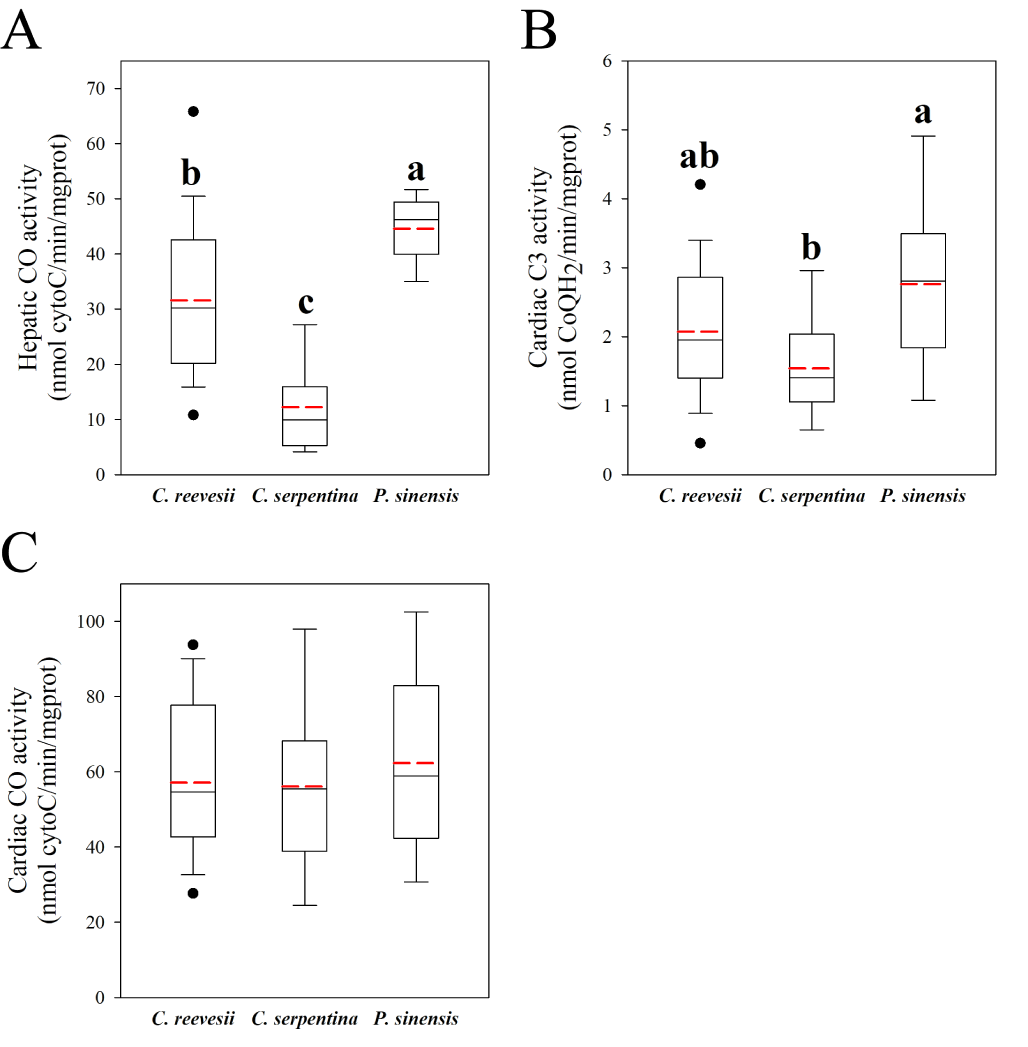

Supplement: Supplementary file 2 — Figure S1. Hepatic CO activity (A), cardiac C3 activity (B) and cardiac CO activity in three freshwater turtle species. Superscripts without common letters denote significant difference (P < 0.05). (TIF 775 kb) [file 12983_2019_301_MOESM2_ESM.tif]

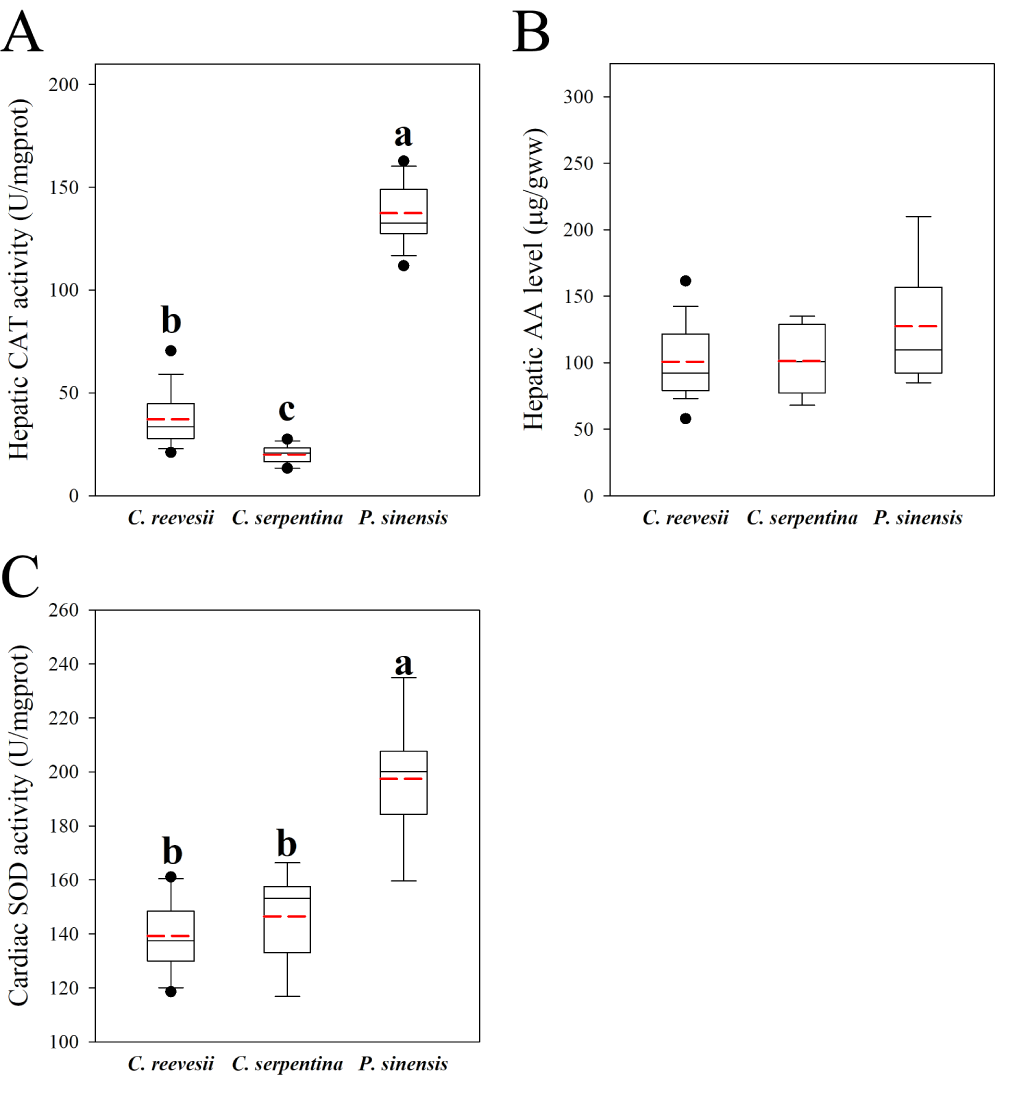

Supplement: Supplementary file 3 — Figure S2. Hepatic CAT activity (A), hepatic AA level (B) and cardiac SOD activity (C) in three freshwater turtle species. Superscripts without common letters denote significant difference (P < 0.05). (TIF 776 kb) [file 12983_2019_301_MOESM3_ESM.tif]

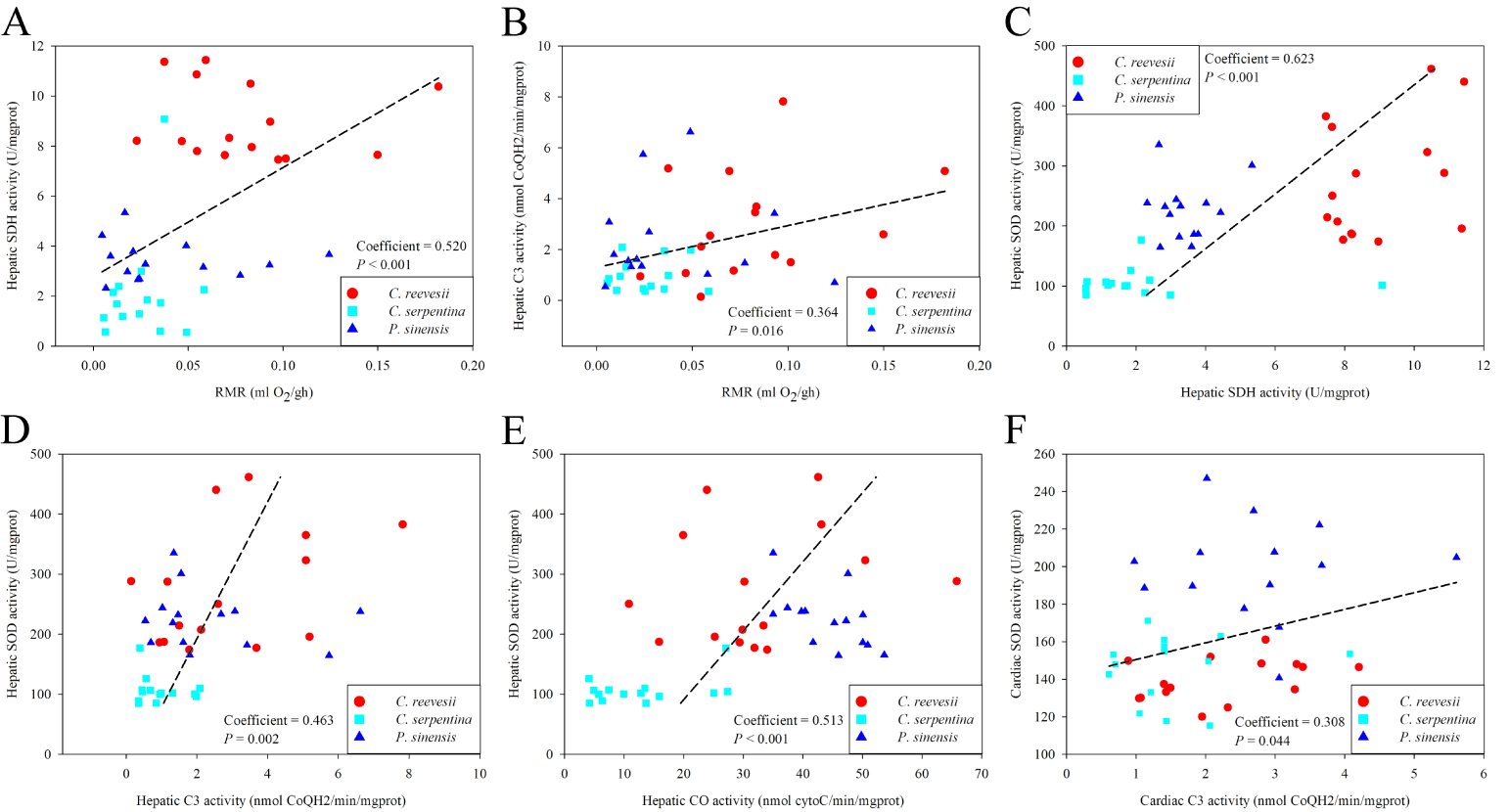

Supplement: Supplementary file 4 — Figure S3. The correlation between parameters measured: hepatic SDH activity and RMR (A), hepatic C3 activity and RMR (B), hepatic SOD activity and hepatic SDH activity (C), hepatic SOD activity and hepatic C3 activity (D), hepatic SOD and hepatic CO activity (E), cardiac SOD activity and cardiac C3 activity (F). (TIF 870 kb) [file 12983_2019_301_MOESM4_ESM.tif]
